# Clinical management of fever in children in Brazil: practical recommendations from an expert panel

**DOI:** 10.31744/einstein_journal/2022RW6045

**Published:** 2022-08-02

**Authors:** Hany Simon, Marcello Creado Pedreira, Silvia Maria de Macedo Barbosa, Tadeu Fernando Fernandes, Ana Maria de Ulhôa Escobar

**Affiliations:** 1 Hospital das Clínicas Faculdade de Medicina Universidade de São Paulo São Paulo SP Brazil Instituto da Criança, Hospital das Clínicas , Faculdade de Medicina , Universidade de São Paulo , São Paulo , SP , Brazil .; 2 Hospital Sírio-Libanês São Paulo SP Brazil Hospital Sírio-Libanês , São Paulo , SP , Brazil .; 3 Faculdade de Medicina Universidade de São Paulo São Paulo SP Brazil Faculdade de Medicina , Universidade de São Paulo , São Paulo , SP , Brazil .

**Keywords:** Fever, Health knowledge, attitudes, practice, Ambulatory care, Child

## Abstract

The objective of this study was to answer several questions related to the assessment and treatment of fever, as well as other controversies that exist during its management in pediatric patients. First, an advisory board with medical experts was conducted to discuss the clinical journey of these patients, considering the main challenges and possible solutions. After this discussion, a non-systematic literature review was performed, between November 2019 and January 2020, to collect the most relevant evidence available in the scientific databases MEDLINE, Lilacs, and SciELO. A narrative review was carried out based on scientific evidence and on extensive experience of experts in clinical practice. The experts developed a set of recommendations and clarifications about the assessment of the severity of fever in pediatrics, the need for treatment and the choice of the most appropriate antipyretic. The most common controversies in the management of fever in pediatric patients were also addressed, such as alternating antipyretics, persistent fever, and dose equivalence. In primary management of pediatric patients, fever should be seen as a relevant symptom that requires treatment with antipyretics in potentially more complex or severe cases, when it causes discomfort to children or is associated with infectious diseases.

## INTRODUCTION

Fever is an increase in body temperature occurring as a result of a systemic response mediated by the central nervous system (CNS). ^( 1 )^ This systemic response may be triggered by several causes, with highlight, in pediatrics, to infectious diseases. Aggressors (viruses, bacteria, fungi, or parasites) activate immune defense mechanisms that, in response, produce cytokines (endogenous pyrogens), such as interleukin (IL) 1, IL-6, tumor necrosis factor (TNF), interferon alpha and beta (IFN-α and β) and the macrophage inflammatory protein (MIP)-1α. ^( 1 )^

The temperature regulating center acts as a thermostat. Normal body temperature is usually between 36.6 and 37.2°C. ^( 2 )^ Body temperature is generally controlled and maintained within this range, despite normal variations in environmental temperature and those physiologically determined by age, time of day, physical exercises or menstrual cycle phase, among other possibilities. ^( 2 )^ When the set point changes upwards, *e.g.,* to 39°C, the body understands that its temperature is low and is propelled to produce heat. ^( 2 )^ Thus, a series of neuroendocrine events are initiated, aiming to increase body temperature. ^( 2 , 3 )^ Usually, an increase by 1°C in body temperature induces a 10% increase in basal metabolism.

Fever is different from hyperthermia. In fever, there is an increase in the hypothalamic set point due to the release of endogenous pyrogens and a series of neuroendocrine events that, in response, increase body temperature. ^( 2 )^ In hyperthermia, there is no elevation of the hypothalamic set point. The increase in body temperature is due to imbalance of the heat-producing and dissipating mechanisms. ^( 2 )^ The causes of hyperthermia may be related to increased heat production, decreased heat dissipation or direct insult to the hypothalamus. ^( 2 )^ Since there is no hypothalamic set point change in hyperthermia, there is no indication for antipyretics.

Currently, fever is one of the most worrying symptoms for parents and caregivers, given the possibility of a child presenting a potentially serious and fatal disease. Seeking health services in the first febrile episode is frequent, since contemporary scientific knowledge indicates that, in several clinical situations, the earlier the diagnosis, the better the prognosis. ^( 4 )^ The source of infection is generally identified by clinical examination or laboratory tests, within up to three days after onset of the first febrile episode. ^( 5 , 6 )^ Once the source of infection has been identified, treatment targeted at the underlying causes is initiated, as soon as possible. Symptomatic relief treatment is usually indicated, since it reduces discomfort for the child.

However, in 20% of febrile events, within the first 7 days, the fever-generating source is not identified by clinical or laboratory exams. ^( 5 )^ This is known as fever without localizing signs (FWLS). Most children with FWLS have an acute, self-limited infectious disease whose etiology is not always identified. An important indicator that can assist in the management of these cases is the overall condition of the child during the fever process. For example, children with temperature of 38°C may present a higher risk of serious illness if they are in depressed general state than children with 39°C who are well disposed. ^( 5 )^ Mintegi et al. pointed out that, in children with up-to-date vaccination status, the possibility of hidden bacteremia decreases significantly, to about 1.6% to 1.8%. ^( 7 )^ Therefore, in children with FWLS with a preserved general state and up-to-date vaccination, it is important that the doctor conduct regular physical examinations and, if deemed needed, indicate collection of relevant laboratory samples, according to the current protocols in force at their organization. ^( 5 )^ It is also important to note that attenuating the child’s physical discomfort and providing the necessary explanations to parents and caregivers, so that they feel emotionally reassured, is fundamental in the FWLS process. ^( 5 )^

To answer several questions related to the evaluation, need for treatment and some controversies that exist in the management of pediatric patients.

Initially, a meeting of medical experts with extensive clinical experience in primary management of fever in children conducted. The group, composed of five experts, thoroughly addressed all the considerations involved in the journey of the pediatric patient with fever.

Subsequently, the most relevant topics for discussion were defined and a non-systematic literature review was carried out between November 2019 and January 2020, to collect the most recent evidence available in databases MEDLINE ^®^ , Latin American and Caribbean Health Sciences Literature (LILACS), as well as in Scientific Electronic Library Online (SciELO). Based on scientific evidence and clinical experience, the experts prepared a set of recommendations and clarifications for the primary management of fever in children. Table 1 summarizes the main topics and recommendations presented in this article.

**Table 1:** Main topics and recommendations

Topics	Recommendations
Severity rating	The existence of a triage scale to standardize pediatric care is a top priority PAT is a fast and ideal approach tool for emergency situations, which helps determine the type of severity of the physiological problem and the priority of early treatment The presence of infection should be investigated if any physiological parameter is found abnormal in PAT. In the case of a normal PAT, the age group and temperature level are considered as parameters to assess the risk of severe bacterial disease
Need for treatment	Fever should be treated when there is significant discomfort for the child, potential or underlying severe disease, or in case of febrile seizures. Other clinical situations must be evaluated according to the general state, age group and temperature level of the child
Management of fever	The use of physical methods is not recommended, and their effect is limited and transient for the treatment of fever The combined or alternating use of antipyretics is not recommended, and there is no scientific evidence to support this indication Evidence shows that acetaminophen, ibuprofen and dipyrone have similar safety profiles, and ibuprofen and dipyrone show superior efficacy in fever control than acetaminophen There is no evidence of equivalence between the different possible doses of antipyretics, and the label for each of the drugs should be checked regarding fever control The choice of the most appropriate presentation should take into account the child’s response upon taking the medication, and alternatives to tablets ( *e.g.* oral drops and suppositories) should be considered, whenever necessary

PAT: Pediatric Assessment Triangle.

## WHY TREAT FEVER?

This is an issue that has been under academic study and discussions for decades. ^( 8 - 10 )^ Those who advocate not treating fever argue that the rise in temperature could more consistently activate immune defense cells, which would be beneficial to the patient. ^( 8 - 10 )^ They also argue fever could slow the growth and reproduction of some viruses and bacteria, a fact that is very possibly related to decreased serum iron. ^( 8 - 10 )^

The arguments in favor of treatment claim that fever causes significant discomfort, and this can be detrimental to the child. The discomfort is mainly due to increased metabolism, oxygen consumption, carbon dioxide production and increased cardiac and respiratory frequencies, with greater cardiopulmonary work. ^( 8 - 10 )^ Ward et al. observe that, in a normal child, the picture may not have consequences other than discomfort itself. ^( 3 )^ However, in children with potentially serious diseases ( *e.g.* pneumonia, meningitis or urinary tract infection) or with underlying diseases, the increase in organic demands could be significantly harmful. ^( 3 )^

The possibility of febrile seizure is another fact that usually points in favor of treating the fever. However, it is important to point out that incidence of febrile seizures is higher in children aged between 6 months and 5 years, affecting approximately 2 to 5% of children in this age group. ^( 11 )^ In case of febrile seizures, it is imperative that any possibility of CNS infection be discarded. Simple seizures are generalized tonic-clonic seizures that last less than 15 minutes and can recur.

Complex seizures are longer, usually lasting longer than 15 minutes; focal, and may be followed by post-ictal neurological changes, such as Todd’s paralysis, with a greater chance of recurring within 24 hours. ^( 11 )^ The course of febrile seizures, whether simple or complex, is generally benign, even in case of recurrence. The most relevant risk factors for recurrence are positive family history, prolonged seizure duration, age less than 18 months and fever intensity. ^( 11 )^

## HOW TO ASSESS SEVERITY OF PEDIATRIC PATIENT WITH FEVER?

### Triage systems in pediatric emergency

A large number of patients seek emergency services every day in search of care, resulting in frequent overcrowding of these environments, and jeopardizing the safety and care of patients with severe diseases. Therefore, it is important to prioritize care of these patients for whom delay in assessment and referral to proper health facilities and initiating treatment can lead to an increase in morbidity and mortality. ^( 12 - 20 )^

Currently, there is no standardized tool for routine use in children. One of the difficulties in developing such tools is related to the variability of clinical parameters, according to the different age groups. An additional concern is that, when critically ill, the child clinically stabilizes at first, but then deteriorates rapidly. Thus, the triage tool may not provide sufficient alert in early stages to ensure adequate care. ^( 21 , 22 )^ Recording vital signs is not sufficient to identify critically ill patients in the emergency room. ^( 23 , 24 )^

Different systems are used internationally to determine initial treatment priorities. They range from unstructured classification, based on “own experience”, to risk stratification tools using color scales. ^( 24 , 25 )^ Color scales can have three (traffic light system) or five levels. Some of these tools are used in private organizations without sufficient documentation or reliability. The five-level triage systems are recommended by national and international societies for emergency triage. ^( 24 , 25 )^

### Main pediatric emergency triage scores

The main tools for pediatric triage are: Australasian Triage Scale, ^( 18 )^ Canadian Triage and Acuity Scale, ^( 12 , 20 )^ Manchester Triage System, ^( 17 )^ Emergency Severity Index, ^( 15 )^ Pediatric Early Warning System score (PEWS), ^( 14 , 21 )^ and Pediatric Observation Priority Score. ^( 19 )^ Magalhães-Barbosa et al. published excellent systematic reviews investigating the validation ^( 26 )^ and reliability ^( 27 )^ of pediatric emergency triage systems.

### Pediatric Assessment Triangle

**Figure 1:**
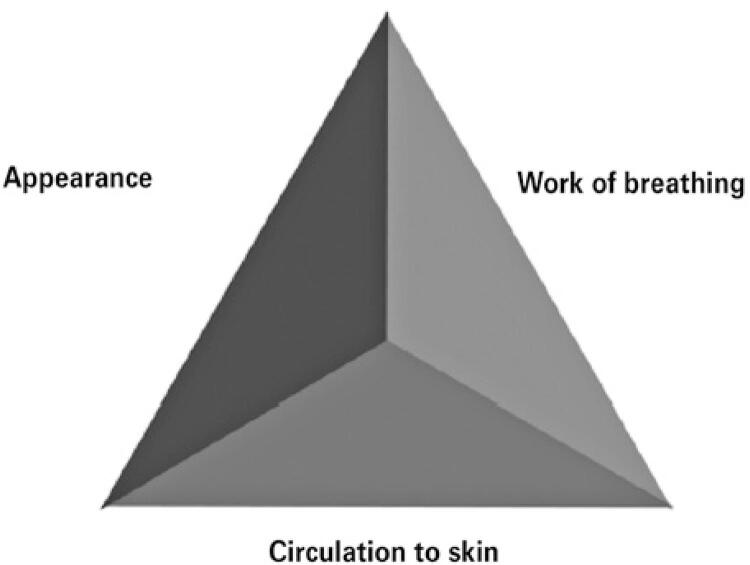
Pediatric Assessment Triangle

Source: Translated and adapted from Dieckmann RA, Brownstein D, Gausche-Hill M. The pediatric assessment triangle: a novel approach for rapid evaluation of children. Pediatr Emerg Care. 2010;26(4):312-5. ^(13)^ ; Duncan H, Hutchison J, Parshuram CS. The Pediatric Early Warning System score: a severity of illness score to predict urgent medical need in hospitalized children. J Crit Care. 2006;21(3):271-8. ^(14)^

In 2000, the American Academy of Pediatrics (AAP) introduced a new fast track tool, the Pediatric Assessment Triangle (TAP) ( Figure 1 ), which allowed physicians to evaluate the overall state of the sick child and establish the severity of clinical presentation, determining the type of emergency intervention. ^( 13 )^ This triangle has become the pillar of pediatric education for pre-hospital care professionals, and has been taught to more than 170 thousand health professionals around the world, having been incorporated into life support courses, such as Advanced Pediatric Life Support (APLS), Pediatric Advanced Life Support (PALS) and Emergency Nursing Pediatric Course (ENPC). ^( 28 )^

The PAT is a fast track tool which takes 30 to 60 seconds to be completed and does not require any devices, only visual and auditory evaluations. The three components of PAT are appearance, working of breathing, and circulation to skin ( Table 2 ). Together, they reflect the general state of oxygenation, ventilation, perfusion, and brain function of the child. ^( 22 , 28 )^

**Table 2:** Characteristics of the three components of the Pediatric Assessment Triangle (appearance, work of breathing and circulation to skin)

PAT component	Normal features
Appearance	
Tone	Moves spontaneously Resists examination Sits or stands (age-appropriate)
Interactivity	Appears alert and engaged with clinician or caregiver Interacts with people, environment Reaches for toys and objects ( *e.g* . a flashlight)
Consolability	Stops crying when held and comforted by caregivers Differential response to caregiver *versus* examiner
Look/gaze	Makes eye contact with the clinician Follows/tracks visually
Speech/cry	Has a strong cry Uses age-appropriate speech
Work of breathing	
Abnormal airway sounds	Snoring, muffled or hoarse speech, stridor, grunting, wheezing
Abnormal positioning	Sniffing position, tripoding, preference for seated posture
Retractions	Supraclavicular, intercostal and substernal retractions, head bobbing (babies)
Flaring	Flaring of the nares on inspiration
Circulation to skin	
Pallor	White or pale skin or mucous membrane coloration
Mottling	Patchy skin discoloration due to varying degrees of vasoconstriction
Cyanosis	Bluish discoloration of skin and mucous membranes

Source: Translated and adapted from: Dieckmann RA, Brownstein D, Gausche-Hill M. The pediatric assessment triangle: a novel approach for rapid evaluation of children. Pediatr Emerg Care. 2010;26(4):312-5; ^(13)^ Duncan H, Hutchison J, Parshuram CS. The Pediatric Early Warning System score: a severity of illness score to predict urgent medical need in hospitalized children. J Crit Care. 2006;21(3):271-8. ^(14)^

PAT: Pediatric Assessment Triangle.

Pediatric Assessment Triangle has the potential to be an optimal screening tool, because it can be applied easily and quickly to stratify stable and unstable patients at different levels of care. ^( 22 )^ In addition, it promotes an initial evaluation of circulatory, respiratory, cerebral and metabolic functions. Each PAT component is evaluated separately, comprising physical, visual and auditory findings ( Table 2 ). If the clinician detects an abnormal finding, the corresponding component is deemed abnormal. This identifies the type of severity of the physiological problem and the priority for initial treatment. ^( 28 )^ The combination of the three PAT components makes up the general impression of the clinician regarding the physiological state of the child, classifying patients as sick or not sick.

## EVALUATION OF CHILDREN WITH FEVER

There are three parameters that are important in the evaluation of children up to 36-month-old with fever: evaluation of the general state, age group and temperature. A child presenting with some degree of impaired general state should be considered as high-risk for severe bacterial disease (SBD). ^( 29 - 31 )^

However, fever can be the only sign of infection in infants with severe bacterial infection, and clinical evaluation alone often fails to identify such patients. ^( 32 )^ Febrile infants aged 60 days or younger, with a temperature greater than 38°C, have between 8% and 13% chance of having a severe bacterial infection, which includes meningitis, urinary tract infection (UTI) and bacteremia. ^( 33 )^ Even if these infants do not maintain their physiological abnormalities, they should be submitted to laboratory investigation of the etiology of fever, and should receive appropriate treatment. ^( 16 , 34 , 35 )^ The prevalence of UTI in children aged 3 to 36 months ranges from 2% to 5%, but there are certain groups in which this risk is higher: white girls less than 2 years old, fever greater than 39 ^o^ C and no apparent source of fever, ^( 36 )^ and uncircumcised boys with fever above 39 ^o^ C and absence of any apparent source of fever. Children who meet high-risk criteria for UTI must be subjected to urine testing. ^( 36 )^ In children aged 3 to 36 months, in addition to the risk of urinary infection, the risk of invasive pneumococcal infection should be assessed. If the fever is greater than 39°C and vaccination ^( 37 - 39 )^ for pneumococcus is incomplete, the child must be fully investigated regarding risk of infection by this agent. ^( 29 - 31 )^ The epidemiology of bacterial infections in children has changed with the introduction of *Haemophilus influenzae* type B vaccines, ^( 40 , 41 )^ and pneumococcal vaccines: PCV7, PCV10, or PCV13. ^( 41 - 45 )^

## HOW TO TREAT FEVER?

### Non-drug treatment

Despite the advances in understanding of pathophysiology of fever and pharmacology of antipyretic drugs, throughout the world, including in Brazil, ^( 46 )^ the reduction of body temperature by physical cooling measures, with cold baths, sponges soaked in cold water and/or alcohol, cooling of the environment, among other anecdotal measures, is still practiced. ^( 47 )^ The physiology of homeothermia is well established; when a physical cooling method is applied, the peripheral temperature decreases by conduction, convection and evaporation methods, but, on the other hand, there is an immediate cardiometabolic response aimed to maintain the body temperature, which induces a rebound effect of temperature elevation. ^( 46 )^

A meta-analysis by Meremikwu et al. included seven studies comparing physical methods to reduce fever with the use of antipyretics or placebo. ^( 48 )^ Some studies highlight the rapid onset of action of these so-called “physical” methods; however, the duration of the desired effect of fever reduction is rapid, with a risk of rebound effects, thus not offering significant advantages over drug interventions. ^( 48 )^

The guidelines of the National Institute for Health and Care Excellence (NICE) do not recommend physical methods to treat fever. ^( 49 )^ The guidelines of the Italian Pediatric Society discourage the use of physical methods because they are not beneficial for children with fever, considering their effects are limited, transient and do not interfere with the body’s central mechanisms of temperature control. ^( 50 )^ The only indication for physical methods is restricted to cases of hyperthermia, a condition for which antipyretics are considered inadequate. ^( 46 )^

### Drug treatment

Fever is a symptom common to several diseases and can cause distress, anxiety and even phobia in parents, causing them to quickly seek to lower their children’s temperature with antipyretic drugs. ^( 51 )^ The most appropriate time for administration and the expected results are still open for discussion, including the fact that the action profile of the antipyretics available is different. ^( 46 )^ One study showed that half of parents do not give the correct dose of antipyretics to their children, which supports the importance of pediatricians being well-informed. ^( 52 )^ In Brazil, data from one study were even more worrying, showing that almost 77% of parents used the wrong dose of dipyrone, ranging from 7.5 to 48.5mg/kg/dose. ^( 53 )^ It is therefore essential that pediatricians know everything about the doses, intervals and expected effects of each drug product, to properly educate families. ^( 52 )^

Table 3 shows a summary of the main characteristics of the three most commonly used drugs for pain and fever management in our setting: dipyrone, a pyrazolone derivative; ibuprofen, a nonsteroidal anti-inflammatory derived from propionic acid; and acetaminophen, a drug of the non-opioid analgesic class. ^( 54 , 55 )^ Acetylsalicylic acid is no longer used in children with fever due to the risk of metabolic disorders. ^( 56 )^ Since the increase in temperature is accompanied by a reduction in blood volume, there could be a relative increase in the concentration of acetylsalicylic acid, leading directly to a metabolic acidosis or respiratory alkalosis with compensatory metabolic acidosis. ^( 56 )^ Other reasons not to use acetylsalicylic acid are potential urticaria reactions and bronchospasm in atopic children, in addition to the risk of Reye syndrome in children with varicella or flu. ^( 56 )^

**Table 3:** The main characteristics of the most widely used analgesic and antipyretic drugs in primary management of fever

Drug	Indications	Pharmacokinetic parameters after oral doses	Pharmacodynamic parameters	Probable mechanisms of antipyretic action	Age and weight ranges approved for use	Main contraindications ^(57-59)^	Adverse effects	Antipyretic dosage recommended in children	Pediatric formulations currently available in the market ^(15)^
Dipyrone	Analgesic Antipyretic	Pro-drug metabolized in the gastrointestinal tract. ^(57)^ Major active metabolite: 4-MAA. ^(54,57)^ Bioavailability of 4-MAA: 89% for the solution formulation (pretty close to that of the intravenous and intramuscular routes). ^(54,57)^ No food-drug interactions. ^(57)^ Plasma half-life of 4-MAA: 2.70.5 hours. ^(57)^ Excretion: 96% in urine and 6% in feces ^(57)^	The metabolite 4-MAA is mainly responsible for clinical effects. ^(57)^ Onset of antipyretic action: 30 to 60 minutes. ^(57)^ Approximate duration of action: 4 hours ^(57)^	Peripheral action: reversible inhibition of prostaglandin synthesis, mainly PGE2, by inhibiting COX-1, COX-2 and possibly COX-3, although evidence still not clear. ^(54,57,60)^ Central action: inhibition of COX-1 and prostaglandin synthesis in the central nervous system, including PGE2, a primary fever mediator also produced in the hypothalamic regulatory center after stimulation of endogenous and exogenous pyrogens ^(57,61)^	From 3 months and over 5kg ^(52)^	Hypersensitivity to the pyrazolone class Diseases of the hematopoietic system Severe allergic reactions to other analgesics Porphyria or G6PD deficiency	Anaphylactic shock, Stevens-Johnson syndrome, agranulocytosis (rare, but may last for up to 1 week), pancytopenia, hypotension and urinary retention	Dosing: 10-16mg/kg. ^(52)^ * Intervals: 6-8 hours. ^(49,52)^ Maximum daily dose: limit 4 doses ^(52)^	Drops: 500mg/mL. (1 drop = 25mg). Oral solution: 50mg/mL. Injectable (IM/IV): 500mg/mL. Suppositories: 300mg
Ibuprofen	Mild to moderate pain Fever	Racemic mixture: inactive R isomer is converted into active S isomer Bioavailability: 80%-85%. ^(58,62)^ Reduced absorption in the presence of food. ^(58)^ Plasma half-life: 1- 3 hours. ^(61,62)^ Excretion: mainly urinary ^(58)^	Pharmacologically active isomer is S. Onset of antipyretic action: 15-30 minutes. ^(58)^ Studies have shown the antipyretic effect may be slower than the analgesic effect, and vary according to the age group of the child (faster in ≤1 year-old). ^(62)^ Approximate duration of action: 4-6 hours ^(58)^	Peripheral action: reversible inhibition of prostaglandin synthesis, including PGE2, by inhibiting COX-1 and possibly COX-2. ^(8,10)^ Other mechanisms proposed, although not fully elucidated: chemotaxis inhibition, changes in lymphocyte activity, platelet aggregation inhibition, activation of neutrophils and decreased levels of pro-inflammatory cytokines. ^(58)^	From 6 months and over 5kg ^(58)^	Hypersensitivity to ibuprofen. Active peptic ulcer or gastrointestinal bleeding Children with history of asthma, rhinitis, urticaria, nasal polyp, angioedema, bronchospasm and other symptoms of allergic or anaphylactic reaction triggered by nonsteroidal anti-inflammatory drugs	Headache, dizziness, edema, gastric intolerance, nausea, constipation or diarrhea, pruritus, rash, bruising	Dosing: 5-10mg/kg. ^(58)^ Intervals 6- 8 hours. ^(58)^ Maximum daily dose: 40mg/kg/day (limit 200mg/ dose and 800mg/day) ^(58)^	Oral suspension (drops): 50mg/mL. (1 drop = 5mg). Oral suspension (drops): 100mg/mL. (1 drop = 10mg). Oral suspension: 30mg/mL.
Acetaminophen	Mild to moderate pain Fever	Bioavailability: 85%-98%. ^(59)^ Slower absorption in the presence of food. ^(59)^ Metabolized mainly by the liver (conjugation and oxidation via cytochrome P450 system). ^(59)^ Plasma half-life in children: 1,5-3 hours (approximately 1 hour longer in newborns). ^(59)^ Excretion: urinary, mainly in the form of metabolites ^(59)^	Onset of antipyretic action: 15-30 minutes. ^(59)^ Approximate duration of action: 4-6 hours ^(59)^	Peripheral action: reversible inhibition of prostaglandin synthesis (including PGE2), mainly through COX-2 enzyme inhibition, but with milder action than selective inhibitors. ^(63)^ Central action: direct effect on the hypothalamic regulatory center ^(59,64)^	From newborns and over 3kg ^(58)^	Hypersensitivity to acetaminophen In moderate renal failure, maximum frequency is every 6 hours. In severe renal failure, maximum frequency is every 8 hours	Hepatotoxic with overdoses or prolonged use of high doses	Dosing: 10-15mg/kg. ^(59,64)^ Intervals: 4-6 hours. ^(59,64)^ Maximum daily dose: 50-75mg/kg (limit of 5 doses). ^(59,64)^ Many children or young people with severe diseases have low weight for age. The doses described here are calculated considering mainly weight, not age, to minimize the risk of overdose per age group	Oral solution (drops) 200mg/mL. (1 drop = 13.3mg). Oral suspension: 100mg/mL. Oral suspension: 32mg/mL. Chewable tablets: 160mg

* The recommended dose range in the package information of the reference product registered with the National Health Surveillance Agency ^(65)^ is approximately 10 to 16mg/kg/dose, ^(57)^ similar to that recommended in the international literature (8 to 16mg/kg/dose). ^(54)^ However, in countries such as Brazil, where dipyrone is widely used, doses of 20mg/kg have been used frequently. ^(54)^ Traditionally, 1 drop/kg of dipyrone per dose is given, equivalent to 25mg/kg per dose.

PEG2: prostaglandins E2; COX: cyclooxygenase enzyme; G6PD: glucose-6-phosphate dehydrogenase; IM: intramuscular; IV: intravenous; MAA: metyl amino antipyrine.

## CONTROVERSIES IN FEVER MANAGEMENT

### Should we alternate antipyretics?

Since the mechanism of action of the three antipyretics available in Brazil is inhibition of prostaglandin E2 (PGE2) synthesis, there is no logic for indicating combined or alternating use. ^( 46 )^ However, despite the lack of scientific evidence to justify alternating antipyretic drugs, this practice has become a habit in pediatric practice. ^( 61 )^ Several publications pointed out this poses risks to the patient, due to errors in the dosing and frequency of antipyretics, promotes fever phobia and increases the risks of intoxication. ^( 66 )^

Despite these recommendations, a survey carried out by American researchers showed that this practice is used by more than 50% of pediatricians. ^( 67 )^ In Spain, a similar study found that close to 69% of local experts use it, according to the publication: “with no scientific documentation whatsoever”. ^( 68 )^

The evidence on the efficacy of alternating antipyretics to manage fever is scarce and the differences found are clinically negligible; in addition, most studies conclude that more research is needed. Therefore, there is no scientific support for this frequent pediatric practice. ^( 46 )^

### Persistent fever: what to do?

The main objective in the treatment children with fever should be their comfort: when the focus is not on temperature values, children with high temperatures can be seen playing, while others with lower values can be in a poor general condition. ^( 46 )^ During the febrile process, dehydration occurs, characterized by insensitive losses due to peripheral vasodilation, accompanied by evaporation of water and solutes. ^( 69 )^ Dehydration is associated with low intake due to the fever-causing underlying and the use of antipyretics, which reduce the production of PGE2. ^( 69 )^ This prostaglandin has a vasodilating effect of extreme importance for the maintenance of pre-glomerular resistance, maintaining the glomerular filtration rate, and preserving renal blood flow. ^( 69 )^

### Dose equivalence of antipyretics and side effects

The most commonly used antipyretics are dipyrone, acetaminophen and ibuprofen, with no equivalence between their various possible doses. The doses proposed in the label of each antipyretic agent usually guide their prescription, and the doses proposed by the manufacture should be used. ^( 52 - 54 )^ The equianalgesic doses and side effects of antipyretics are shown in table 3 .

### What to do if the child vomits the medication?

Rejection of unpleasant medication is a physiological reflex of the child. The more bitter and irritant the taste, the greater the probability that the drug will be rejected by a child. ^( 70 )^ Currently, the pharmaceutical market offers good antipyretic formulations with sweetened excipients that mask their taste, in an attempt to increase acceptance. ^( 46 )^ However, when acceptability and viability of oral antipyretics are limited, injectables or suppositories can be useful, especially in children who systematically vomit or reject oral antipyretics. ^( 70 , 71 )^

Currently, there is no scientific evidence supporting repeated administration of the same or other agents in cases of vomiting.

### Does the formulation of the antipyretic make a difference?

The formulation of the antipyretic does make a difference because there is no standardization of the volume and concentration of each drop of the agent. ^( 57 - 59 )^ This occurs mainly with dipyrone and acetaminophen. Therefore, it is difficult to know what dose is being given to a certain patient, since in many cases the manufacturer and concentration of each milligram of the drug are unknown. This represents a confounder in the administration of each drug. For example, dipyrone concentration ranges from 25 to 50mg/mL.

Oral drops are usually more palatable and allow for greater adherence to the prescribed treatment. In Brazil, the oral solution is available for dipyrone and acetaminophen. The only medicine that is available in a rectal formulation is dipyrone. Its use is indicated in patients who, for any reason, cannot receive the medication by another route. Adequate use of the antipyretic, regardless of the formulation, is key for appropriate fever and pain management.

### Which drug of choice (efficacy comparison)?

First, the choice of the antipyretic to be used has always been a cause of controversy in pediatric practice. In 2001, Wong et al. carried out a study comparing the antipyretic efficacy of acetaminophen, ibuprofen and dipyrone (metamizole) in children aged 6 months to 6 years, with fever. ^( 72 )^ The study included 628 febrile children and evaluated the effect of the three antipyretics used. A large number of subjects completed the study (555), all antipyretics investigated had the desired effect; however, in the ibuprofen and dipyrone groups, the temperature normalization rates were significantly better (78% and 82%, respectively), when compared to acetaminophen (68%). After a period ranging from 4 to 6 hours, the mean temperature in the dipyrone group was significantly lower than that of the other study drugs. As for tolerability, the three antipyretics had comparable profiles. ^( 72 )^ Another open, randomized, controlled study carried out in Brazil, in 2011, also investigated 80 febrile children aged between 6 months and 8 years. In this study, Magni et al. compared the antipyretic effect of a single dose of ibuprofen (10mg/kg) and dipyrone (15mg/kg) in two groups of children by fever severity: high fever (>39.1 ^o^ C) and low fever (38.0 ^o^ C to 39.1 ^o^ C). Of the 80 children, 41 received ibuprofen (51.2%) and 39 dipyrone (48.8%). All children (100%) experienced fever reduction 2 hours after treatment. In the high fever group, the temperature reduction was statistically significant with ibuprofen compared to dipyrone, 3 (p=0.007) and 4 hours (p=0.025) after administration. Both drugs had acceptable tolerability profiles during the observation period. ^( 73 )^

It should be noted that these three drugs are not only antipyretics. They all have relevant analgesic efficacy. At the time of prescription, pediatricians should consider the indication of the antipyretic, the age of the child, and the underlying disease of the patient. A rational choice of the drug to be used helps ensure the efficacy and safety of the various medicines to control this symptom.

## CONCLUSION

In the primary management of pediatric patients with fever, there are still several myths and controversies regarding the assessment of severity, the need for treatment and the choice of the most adequate antipyretic agent. In this light, several recommendations were presented for each point of the process, based on scientific evidence and clinical experience. Fever must be seen as a relevant symptom requiring treatment with antipyretics in potentially more severe or complex cases, accompanied by discomfort to the child or associated with infectious diseases.
